# Dementia Care is Relational and Communal: Lessons from Indigenous Communities in Canada and the US

**DOI:** 10.1093/geroni/igaf122.168

**Published:** 2025-12-31

**Authors:** Dana Ketcher, Elizabeth Weigler, Margaret Noun, Melissa Blind, Nickolas Lambrou, Megan Zuelsdorff, Carey Gleason, Kristen Jacklin

**Affiliations:** University of Minnesota Medical School, Duluth Campus, Duluth, Minnesota, United States; University of Minnesota Medical School, Duluth Campus, Duluth, Minnesota, United States; University of Minnesota Medical School, Duluth Campus, Duluth, Minnesota, United States; University of Minnesota Medical School, Duluth Campus, Duluth, Minnesota, United States; University of Wisconsin–Madison, Madison, Wisconsin, United States; University of Wisconsin Madison, Madison, Wisconsin, United States; University of Wisconsin–Madison, Madison, Wisconsin, United States; University of Minnesota Medical School, Duluth Campus, Duluth, Minnesota, United States

## Abstract

This paper presents findings from the Indigenous Cultural Understandings of Alzheimer’s Disease and Dementia (ICARE) Project. We share our analysis of qualitative data generated in partnership with four Indigenous communities in Canada and the US. We interviewed dementia program and health care administrators (n = 20) and held sequential focus groups with local health care staff/formal caregivers who work with Indigenous older adults (18 sessions, n = 17) between 2018-2021. Topics focused on the community experience of dementia across the disease trajectory. Four qualitative analysts coded and analyzed data in partnership with PIs, community-based researchers, and senior researchers. The analysis resulted in identifying three key cultural responses communities employ to care for people living with dementia across the disease continuum. First, a relationality perspective that underscores the importance of ongoing care needs rooted in relationship-building through cultural practices such as visiting. Second, community care activates community support systems, prioritizing training opportunities and decentralizing responsibilities and rights to care away from Western institutions. Finally, care models that support and center the family; coordinating with family members across institutions and specialists were a key concern. These findings have been translated into an Indigenous dementia care framework that highlights these three domains and prioritizes strength-based strategies that communities use to mitigate the colonial erasure of Indigenous practices and worldviews. Working with community partners, we have received further feedback on the framework, identifying differences in community uses for the framework and issues that apply more broadly across the four sites for future work.

